# Functional Ingredients and Additives from Lemon by-Products and Their Applications in Food Preservation: A Review

**DOI:** 10.3390/foods12051095

**Published:** 2023-03-04

**Authors:** Daniela Magalhães, Ana A. Vilas-Boas, Paula Teixeira, Manuela Pintado

**Affiliations:** CBQF—Centro de Biotecnologia e Química Fina—Laboratório Associado, Escola Superior de Biotecnologia, Universidade Católica Portuguesa, Rua Diogo Botelho 1327, 4169-005 Porto, Portugal

**Keywords:** citrus waste, lemon by-products, high-biological-value compounds, essential oils, phenolic compounds, dietary fibres, circular economy, food preservation

## Abstract

Citrus trees are among the most abundant fruit trees in the world, with an annual production of around 124 million tonnes. Lemons and limes are among the most significant contributors, producing nearly 16 million tonnes per year. The processing and consumption of citrus fruits generates a significant amount of waste, including peels, pulp, seeds, and pomace, which represents about 50% of the fresh fruit. *Citrus limon* (*C. limon*) by-products are composed of significant amounts of bioactive compounds, such as phenolic compounds, carotenoids, vitamins, essential oils, and fibres, which give them nutritional value and health benefits such as antimicrobial and antioxidant properties. These by-products, which are typically discarded as waste in the environment, can be explored to produce new functional ingredients, a desirable approach from a circular economy perspective. The present review systematically summarizes the potential high-biological-value components extracted from by-products to achieve a zero-waste goal, focusing on the recovery of three main fractions: essential oils, phenolic compounds, and dietary fibres, present in *C. limon* by-products, and their applications in food preservation.

## 1. Introduction

**Figure 1:**
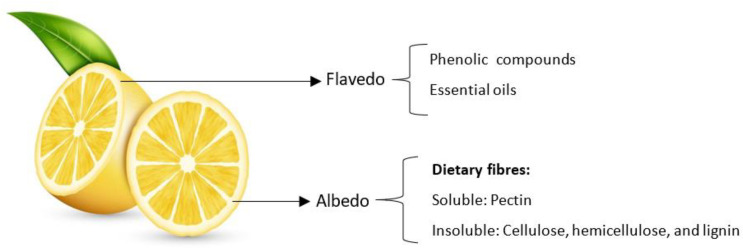
Schematic view of the main structural composition of *C. limon* peels.

The genus *Citrus* belongs to the family *Rutaceae*, and includes one of the most widely consumed fruits in the world [1,2]. It represents an annual production of approximately 124 million tons, of which the most important are oranges, *Citrus sinensis* L. (67 million tons); mandarins, *Citrus reticulata* L. (33 million tons); lemons, *Citrus limon* L.; and limes, *Citrus aurantifolia* L. (16 million tons) [3]. The major producers of *C. limon* are India (3 million tons), China (2 million tons) and Mexico (2 million tons) [3]. Fresh production and processing generate a huge amount of waste, such as peels, seeds, and pulps, which represent about 50% of the raw fruit and are potential sources of added-value co-products. Although some have been valorised, other residues are still disposed of in landfills, causing environmental problems due to the molecules present in the peels, especially lipids, carbohydrates, and phenolic compounds, which also constitute a relevant loss due to their bioactive value. Valorising these by-products increases the potential economic return while at the same time helping to preserve the environment [4,5,6]. Lemon is one of the most important crops in fruit production and is widely used in beverages, ice creams, and desserts, and as cooking ingredients, due to its tart flavour. Industrialization of lemon processing creates large quantities of by-products, which can be of interest to the food industry due to their high levels of nutrients and bioactive compounds (BCs), such as polyphenols, carotenoids, vitamins, essential oils (EOs), and dietary fibre, including pectin, cellulose, hemicellulose, and lignin [7]. Previous studies have shown that lemon peel (LP) contains higher levels of phenolic compounds and dietary fibres than the flesh [8]. Structurally, the LP consists of an outer layer, called the epicarp or flavedo, and an inner layer, called mesocarp or albedo [9]. The flavedo contains high amounts of phenolic compounds, particularly flavonoids: hesperidin, diosmin, eriocitrin, and narirutin, whereas the albedo is rich in fibres, particularly pectin [10] (Figure 1).

**Figure 2:**
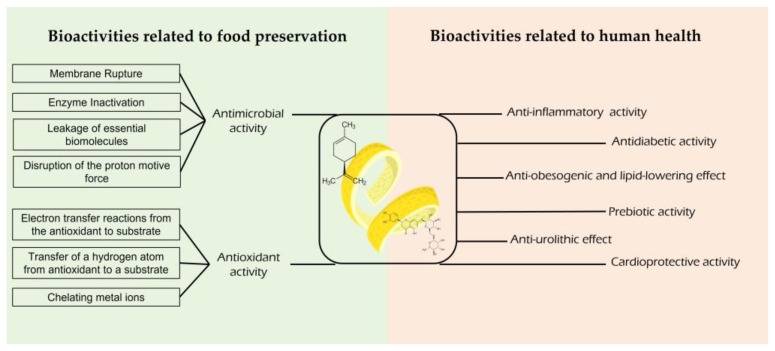
Outline of the main bioactivities of lemon by-products related to food preservation and human health with focus on the mechanisms of action of antimicrobial and antioxidant activity.

These compounds often impart health benefits, including antioxidant, antimicrobial, anti-inflammatory, prebiotic, cardioprotective, anti-obesogenic, and lipid-lowering benefits, among others. The bioactivities of these BCs most related to food preservation are antimicrobial and antioxidant activities (Figure 2). The antimicrobial properties are mainly attributed to the lemon EOs and show some different mechanisms of action, including (i) membrane rupture with inhibition ATP-ase activity, (ii) leakage of essential biomolecules from the cell, (iii) disruption of the proton motive force, and (iv) enzyme inactivation [11]. The biological activities of phenolic compounds are well documented, and act by changing cell permeability, allowing the loss of essential biomolecules, so that the membrane loses its structure and functionality [4,12,13]. Antioxidant activity is more characteristic of phenolic compounds; however, several studies report the potential of EOs in this context. The antioxidant mechanism is divided into three main mechanisms: (i) electron transfer from the antioxidant compound, (ii) hydrogen atom transfer from the antioxidant compounds and (iii) chelation of metal ions [14].

Citrus EOs are among the most noteworthy by-products of citrus processing. These are already being extracted by industries and can be used as natural additives and preservatives in several food and beverage products [13]. The use of EOs in cosmetic products is also common. These components have gained acceptance in the food industry, since they were generally recognized as safe (GRAS) by the US Food and Drug Administration (US FDA) [15,16], and some studies show that certain foods tolerate their presence [17,18]. EOs could represent a ”natural” alternative to the synthetic antioxidants and antimicrobials in foods, and consequently, consumers and food producers are increasingly interested in EO applications [19]. However, the use of EOs in food preservation is often limited by the costs of application and other drawbacks such as strong off-flavours and potential toxicity. Incorporating these compounds into the formulation of edible coatings could be a solution to reducing the concentration at which they are applied without losing their efficacy [20]. There are some review articles focused on the potential of bioactive lemon fractions for industrial and nutraceutical purposes [10,21,22]; however, there are gaps regarding the use of these fractions in food preservation. For this reason, this study aims to systematically review recent scientific works related to the valorisation of lemon by-products to produce new functional ingredients/additives with high-biological-value: EOs, polyphenols, and pectin, and their related applications in food preservation.

## 2. Studies Published in the Last Two Decades—An Overview

### 2.1. Research Methodology

Scientific publications on lemon by-products published in the past two decades were obtained through a search in Web of Science using the keywords “lemon peels” and “lemon by-products” and selecting the options “all fields”. This means that all papers which were available in the Web of Science databases and that had the words “lemon peels” and “lemon by-products” in their title, abstract, or keywords were included. The abstracts of all papers were analyzed to classify them into one of five categories (Essential oils: extraction and characterization; Phenolic compounds: extraction and characterization; Pectin: extraction and characterization; Bioactive properties; and Food applications. Abstracts of meetings and patents, and papers not written in English or where lemon was not analyzed as a by-product, were eliminated.

### 2.2. Results

**Figure 3:**
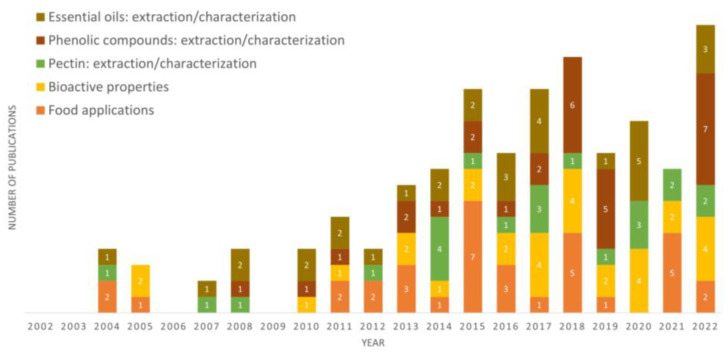
Number of research papers related to lemon by-products published in the last two decades. The search was carried out in Web of Science, using the keywords ‘’lemon by-products’’ and “lemon peels”. All abstracts were analyzed to classify the papers in one of five categories: essential oils: extraction/characterization; phenolic compounds: extraction/characterization; pectin: extraction/characterization; bioactive properties; and food applications.

Figure 3 shows the number of research papers on lemon by-products published over the last two decades. According to our search, 147 papers have been published since 2002. There is an increasing trend in the number of publications over the years (2002–2008: 13; 2009–2013: 22; 2014–2017: 47; 2018–2022: 65), which shows that the valorisation of lemon by-products is a growing area of research. The nutritional and phytochemical characterization of lemon by-products has been extensively studied. Concerning studies about the extraction of bioactive fractions from lemon by-products, most of them had the recovery of essential oils and phenolic compounds as the main goal. Overall, the most-studied bioactive properties of lemon peels are the antimicrobial and antioxidant activities. However, more research studies on the bioactivities of recovered bioactive fractions from lemon by-products are needed, especially regarding pectin bioactivities. Concerning food applications, the incorporation of lemon peel powders or lemon fractions into food products has been studied since 2004. Furthermore, the application of essential oils, extracts, or pectin from lemon by-products in the development of edible coatings has been evaluated in recent years.

## 3. Lemon by-Products: Value-Added Compounds

### 3.1. Volatile Compounds/Essential Oils

#### 3.1.1. Composition

The composition of the EOs is variable, depending on several factors such as geographic location, climate, species, maturity level, and extraction method [23]. Lemon EOs have a natural and safe fragrance and high nutritional and medicinal value, which can enhance the circulatory system and boost immunity, and prevent diseases such as depression, anxiety, and neurological disorders. EOs are important components of the flavour quality of lemon fruits, where 85–99% (*m*/*m*) of the EOs are composed of volatile flavour compounds: monoterpenes, sesquiterpenes, aldehydes, monoterpene alcohols, and monoterpene esters [24]. Limonene, the main compound of lemon EO, has the GRAS status recognized by the US FDA. Oral toxicity studies of limonene have been conducted in mice and rats. In agreement with the European Food Safety Agency (EFSA), the No Observable Adverse Effect Level (NOAEL) was set at the dose of 215 mg/kg/day administered to female rats in the carcinogenicity study [25,26]. Furthermore, limonene exhibits antimicrobial properties, including activity against common postharvest fungal pathogens in fruit [20]. Volatile compounds in lemon by-products have been investigated in recent years (Table 1). Caputo et al. [27] showed the presence of 30 components in lemon EO, identified by GC-MS, accounting for 97.9% of the total EO. The major components were limonene (57.7%), *γ*-terpinene (10.5%), and *β*-pinene (9.3%). Espina et al. [16] identified the same major components: limonene (59.1%), followed by *γ*-terpinene (9.7%) and *β*-pinene (5.2%). Di Rauso Simeone et al. [19] studied four different lemon Italian cultivars at different ripening stages. This study showed the same major compounds as the other studies, but at different levels, depending on the cultivar and ripening stages, where limonene was found in the range of 63.7 to 76.9%. Aguilar-Hernández et al. [28] studied five varieties of lemon: Verna, Betera, Eureka, Fino 49, and Fino 95, and showed that limonene was variable according to the cultivar, ranging from 19.7 g/kg in Verna to 22.7 g/kg in Eureka, followed by *β*-pinene ranging from 3.8 g·kg^−1^ in Fino 95 to 5.0 g/kg in Verna. Among the varieties, the highest concentration of total volatile compounds was found in Eureka, followed by Bétera > Fino 95 > Verna > Fino 49. A recent study by Wang et al. [24] investigated the volatile compounds in Eureka lemon in different by-products: flavedo, albedo, juice vesicles, and segment wall. Limonene is the major volatile compound present in all by-products and flavedo is the by-product with the highest concentration of this compound (14.0 mg/g). According to the results presented in Table 1, there are many similarities between the studies on lemon by-products, and it is also possible to conclude that limonene is the major compound present in EOs extracted from lemon by-products, and that Eureka is the variety with the highest limonene concentration. Therefore, lemon EOs may be a new potential source of natural antimicrobial and antioxidant agents applied in the food industry.

#### 3.1.2. Extraction Technologies and Preservative-Related Properties

**Figure 4:**
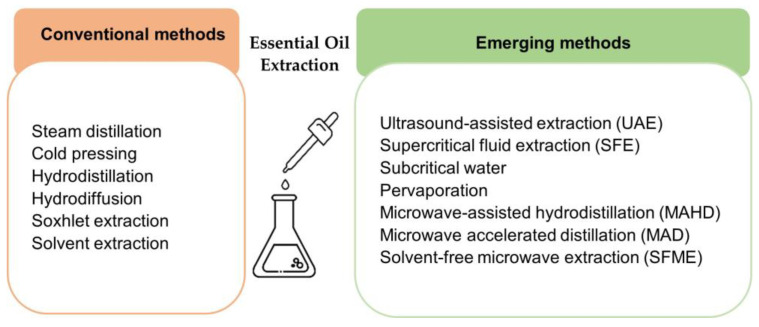
Schematic of essential oil extraction technologies.

Essential oils can be extracted from lemon by-products by various extraction methods, which is one of the prime factors determining their quality. An inappropriate extraction procedure can damage or alter the action of the chemical signature of EOs, resulting in discolouration, off-odor/flavour, and the loss of bioactivity and natural characteristics [36]. Extraction can be carried out by conventional methods such as steam distillation, hydrodistillation, and cold pressing. However, conventional methods show disadvantages, such as higher energy consumption and longer extraction time, which results in higher cost [37]. Steam distillation is generally used at an industrial scale, especially for D-limonene recovery. Unfortunately, besides the disadvantage of low EO removal, the high temperature and the long extraction time involved could modify the volatile molecules. EOs obtained through steam distillation can be easily degraded because of the instability of terpene hydrocarbons [38]. Therefore, the development of green methods having higher extraction efficiency is an area that needs to be explored. In this context, the most recent developments are based on ultrasound-assisted extraction (UAE), supercritical fluid extraction (SFE), and microwave-assisted hydrodistillation (MAHD) [36,39]. Figure 4 shows the conventional and the emerging methods of EO extraction.

The bioactive components of EOs play critical roles due to their antibacterial, antioxidant, and anti-inflammatory activities [29]. Table 2 shows some studies related to lemon oil extraction technologies and their bioactive properties. Ferhat et al. [40] performed a study comparing two conventional methods, hydrodistillation (HD) and cold pressing (CP), with an emerging method, microwave accelerated distillation (MAD). Microwave distillation showed shorter extraction times compared with the other extraction technologies (30 min for MAD vs. 3 h for HD and 1 h for CP) and better yields (0.2% for MAD vs. 0.2% for HD and 0.1% for CP). These EOs showed antimicrobial activity against the six microorganisms tested. The results obtained with HD, CP, or MAD were similar. Moosavy et al. [41] used steam distillation for extracting lemon EOs and tested the antioxidant and antimicrobial activity of these compounds. Lemon peel EO showed 55.1% inhibition of 2,2-Diphenyl-1-picrylhydrazyl (DPPH), and the MIC and MBC values of the EO against *Staphylococcus aureus* were 1.3% and 5%, respectively. Asker et al. [42] used conventional hydrodistillation to extract lemon EOs and proved an inhibition against *S.aureus* and *Pseudomonas aeruginosa.* Golmakani et al. [43] showed that the half maximal inhibitory concentration (IC_50_) values of the EOs extracted by HD, microwave-assisted hydrodistillation (MAHD), and solvent-free microwave extraction (SFME) were 44.1, 42.0, and 97.2 mg/mL, respectively. The lower scavenging activity (higher IC_50_ value) of SFME is related to its lower concentrations of antioxidant compounds such as limonene. Oboh et al. [44] used hydrodistillation for extracting lemon peel EO. This study was designed to evaluate the interactions of EOs from LP on enzymes linked to type-2 diabetes (α-amylase and α-glucosidase) and hypertension (angiotensin-I-converting enzyme [ACE]). LP essential oil exhibited stronger inhibitory activity on α-amylase (IC_50_ = 8.16 µg/mL), α-glucosidase (IC_50_ = 7.56 µg/mL), and ACE (IC_50_ = 26.17 µg/mL). According to the presented studies, this bioactive fraction (with antioxidant and antimicrobial capacity) is a good natural alternative to chemical preservatives and other synthetic antioxidants, such as sodium nitrites, nitrates, or benzoates, commonly utilized in food preservation.

### 3.2. Phenolic Compounds

#### 3.2.1. Composition

Phenolic compounds (PCs) are the most important group of bioactive compounds in both citrus fruit juices and by-products, determining their biological activity. This class of compounds is divided into five main classes: flavonoids, phenolic acids, tannins, stilbenes, and lignans. Flavonoids stand out as one of the largest groups of phenolic compounds, and are present almost in all parts of the plants. These compounds contain two aromatic rings (A & B) bound by a pyrone or hydropyrone ring (C), the flavones or flavanones, respectively [48]. More than 60 individual flavonoids have been identified in *Citrus* sp. In lemon were found the flavanones eriocitrin, hesperidin, naringin, neoeriocitrin, neohesperidin, narirutin, eriodictyol, and naringenin; the flavones diosmetin, diosmin, luteolin, vicenin, chrysoeriol, apigenin, and sinensetin; and the flavonols quercetin, limocitrin, limocitrol, rutin, and kaempferol. Phenolic compounds include phenolic acids such as hydroxycinnamic acids—chlorogenic, caffeic, ferulic, sinapic, and p-coumaric, and hydroxybenzoic acids—protocatechuic, p-hydroxybenzoic, vanillic, and gallic [10]. Some research studies have shown that, in most fruits, peels have a higher content of polyphenols when compared with pulp, which is expected since polyphenols are the main compounds responsible for the protective role against insects and microorganisms. In addition, the PC concentrations in lemon fruits depend on the cultivar, maturity stage, etc. [49]. Table 3 shows the recent studies about the major PCs present in lemon by-products. Gargouri et al. [50] found six phenolic acid derivatives and 32 flavonoids. Regarding the flavonoids, the major class found was flavanones, with a concentration of hesperidin of 1234.8 μg/g DM and eriocitrin of 955.4 μg/g DM. Xi et al. [9] performed a detailed study in five lemon cultivars for different types of by-products: peel, pulp, juice, seeds, and whole fruit. Caffeic acid and chlorogenic acid were the dominant phenolic acids in the tested lemon, varying in the ranges 9.31–741.6 and 2.70–527.5 µg/g fresh weight (FW), respectively, and hesperidin was the predominant flavanone, varying in the range 10.3–3315 µg/g FW. The polyphenol and antioxidant capacities of the different fruit parts were peels > whole fruit > pulp > seed > juice. According to the results presented in Table 3, citrus by-products are a source of valuable phenolic compounds and can be used in food products as active ingredients or additives, as replacements for synthetic preservatives, such as butylated hydroxyanisole (BHA) and butylated hydroxytoluene (BHT), to improve the health-promoting value of the product and respond to the clean label trends. 

#### 3.2.2. Extraction Technologies and Preservative-Related Properties

Phenolic compound extraction in food matrices can be carried out by conventional methods. These methods are characterised mainly by high extraction time, huge energy consumption, and solvent wastage. Thus, the application of these techniques is not aligned with the sustainable development goals (SDGs) adopted by the United Nations to overcome pollution, climate change, misuse of natural and man-made resources, and food security. Conventional extraction methods mainly include maceration, decoction, percolation, infusion, digestion, serial exhaustive extraction, and Soxhlet extraction [56]. These are varied, depending on the composition and characteristics of food samples. Since conventional extraction methods suffer some drawbacks, it is essential to overcome these challenges. This need brought about the use of unconventional extraction methods, which have been created to fill the missing gaps in conventional methods. These methods include pressurized liquid extraction, subcritical water extraction, supercritical fluid extraction, microwave-assisted extraction, solid-phase extraction, ultrasound-assisted extraction, pulsed electric field extraction, high-hydrostatic-pressure extraction, solid-supported liquid-liquid extraction, matrix solid-phase dispersion, and counter-current chromatography [57]. Table 4 shows some studies on phenolic compound extraction technologies and their bioactive properties. Saini et al. [58] compared a conventional extraction (maceration) with a non-conventional extraction (ultrasound-assisted extraction (UAE)) to extract polyphenols and determine their antioxidant activity. A DPPH assay showed that antioxidant activity using maceration was 22.46%, and 39.73% using UAE. UAE is advantageous because of its simplicity, reduced operational time, low toxicity, and minimal energy and solvent consumption. Therefore, the UAE technique has wider applications in the industrial sector than conventional techniques. Peiró et al. [59] showed that an electric field intensity of 7 kV/cm increased the efficiency of polyphenol extraction by 300%, giving maximum values of 84 mg of hesperidin in 100 g FW and 176 mg of eriocitrin in 100 g FW, and the samples with higher phenol content rate showed higher antioxidant activity. These authors conclude that PEF provides a new methodology to improve polyphenol extraction with a non-thermal and environmentally-friendly technology. Dahmoune et al. [60] evaluate the antioxidant activity of polyphenol lemon extracts using conventional solvent extraction (CSE) and two non-conventional methods: ultrasound-assisted extraction (UAE) and microwave-assisted extraction (MAE). From the point of view of the antioxidant activity, the DPPH test showed that MAE was the best technology due to the significantly lower IC_50_ (203.59 ± 5.59 g GAE/mL) than that obtained UAE (268.24 ± 10.62 g GAE/mL), which was, in turn, significantly lower than that obtained by CSE (298.82 ± 8.60 g GAE/mL. The proposed MAE method appeared to be better than both UAE and CSE, allowing for higher recovery polyphenol yield and specific antioxidant activity with a shorter working time and a lower solvent consumption. Some in vivo studies were performed regarding PCs’ bioactive potential. Zou et al. [61] investigated the effect of a lemon-peel extract in a rat model of rheumatoid arthritis and SW1353 chondrocytes. The major PCs found in the LP extract were pyrogallic acid, salicylic acid, luteoline, and p-coumaric acid, among others. The extract was administered orally for five consecutive weeks, and it was observed that the LP extract ameliorated rheumatoid arthritis, and reduced the SW1353 chondrocyte proliferation, ROS, inflammatory cytokine, and xanthine oxidase levels. Gao et al. [62] investigated the protective effect of LP polyphenols (LPP) on human keratinocyte HaCaT cells under oxidative stress. Cell survival rates were determined by MTT assay, and the antioxidant enzyme activity and antioxidant activity of cells were determined using a kit. LPP significantly protects HaCaT cells against oxidative damage. The mechanism of this effect appears to be through regulation of the Nrf2/HO-1 signaling pathway by the eight identified active substances (protocatechic acid, caffeic acid, neochlorogenic acid, (+)-catechin, gallic acid, (−)-catechin gallate, isochlorogenic acid, and rosmarinic acid), resulting in improvement of antioxidant enzymes such as SOD, GSH, and CAT in skin cells, and inhibition of apoptosis.

### 3.3. Dietary Fibre/Pectin

#### 3.3.1. Composition

Lemon fruits are considered a rich source of dietary fibres (DF), which are classified into soluble (SDF) and insoluble dietary fibres (IDF) based on their solubility in water. Examples of SDFs include gums, pectin, glucans, and some biological and synthetic polysaccharides, while IDFs include cellulose, hemicellulose, and lignin [67]. The content of all dietary fibre fractions (total, soluble, and insoluble) is higher in peels (ca. 65%) than in peeled citrus fruit, in particular pectin, which is the major component of fibre present in lemon fruit [22,68]. Pectin provides structural integrity, strength, and flexibility to the cell wall and a barrier to the external environment. Humans are not able to digest pectin due to its resistance to the digestive system and the lack of pectin digestive enzymes. However, microorganisms present in the large intestine can assimilate the pectin and convert it into beneficial metabolites. Therefore, these metabolites promote a positive impact on gut microbiota [69]. Furthermore, the antimicrobial activity of pectin is one of the most relevant claimed bioactivities [70]. However, studies proving the antimicrobial activity of pectin extracted from lemon by-products are still scarce. A reasonable dietary fibre intake recommended is 25–30 g/day and *C. limon* may constitute a valuable contribution to meeting the daily fibre requirement [71]. The main advantage of dietary fibre from citrus fruit when compared to alternative sources of fibre, such as cereals, is its higher proportion of SDF, with about 33% in citrus fruits. In comparison, only 7% is present in wheat bran. This is an important point, considering that the requirement for dietary fibre intake must be well-adjusted, i.e., the water-soluble fraction should represent between 30 and 50% of the total dietary fibre. In addition, citrus fruits have a better quality of DF than other sources of DF due to the presence of associated bioactive compounds with antioxidant properties, which may exert stronger health-promoting effects than the dietary fibre itself [72]. Table 5 shows the recent studies on the dietary fibre composition of lemon by-products. Czech et al. [51] evaluated the content of total dietary fibre in peel, pulp, and whole fruit, and observed that peels contain the highest concentration of fibre (64.64 g/100g). Also, Fernández-López et al. [68] reported that peels (2.49 g/100g of FW) contained a higher concentration of fibre than peeled lemon (1.31 g/100 g of FW). For both, insoluble fibre represented 67% and soluble fibre represented 33%. Martin et al. [73] performed a detailed study of lemon peels and pulp provided from a commercial orchard. The peels contained 13% DW of pectin, 7.56% DW of lignin, 23.06% of cellulose, and 8.09% of hemicellulose. The pulp contained 22.53% DW of pectin, 7.55% DW of lignin, 36.22% DW of cellulose, and 11.05% DW of hemicellulose. In this study we can observe that the pulp contained a higher content of pectin, cellulose, and hemicellulose compared with peels, and that IDF represented most of the fibre for both peels and pulp. However, it is important to notice that pectin (soluble fibre) is a very relevant source of fibre. Figuerola et al. [74] compared the dietary fibre content in two lemon varieties: Eureka and Fino 49. The peels had a higher value of total dietary fibre in Fino 49 (68 g/100g DW) compared with Eureka (60.1 g/100g DW). According to the results presented in Table 5, it is possible to conclude that lemon by-products are good sources of dietary fibres, especially cellulose (insoluble fibre) and pectin (soluble fibre). These dietary fibres can be used as functional ingredients/additives to improve food products’ quality and nutritive properties.

#### 3.3.2. Extraction Technologies and Preservative-Related Properties

The current industrial extraction of pectins from lemon by-products generally employs mineral acids to lower the pH value (1.2–3) at high temperatures (60–100 °C) and during long periods (20 to 360 min). After the extraction, the process includes precipitation with alcohol (ethanol, isopropanol, or methanol) and purification with dia-ultrafiltration and concentration using a fiber membrane [77]. However, this approach is no longer environmentally sustainable since the use of mineral acids can be harmful to the environment. Therefore, in recent years, the research community showed that replacing mineral acids with organic acids such as citric acid could be a sustainable alternative to extracting pectin with higher yield quality in comparison with the conventional method [78]. In addition, other novel green technologies (microwave, ultrasound, high pressure, hydrodynamic cavitation, subcritical water, enzyme utilization, and electromagnetic induction heating have been reported as sustainable alternatives to obtain pectin from several by-products [79,80]. Traditionally, pectin is a hydrocolloid commonly used in the food industry as a texturizer, stabilizer, and thickening agent in jams, low-calorie soft drinks, and dairy and meat products [81]. The ability of pectin to form a gel depends on molecular size and degree of esterification (DE). According to the number of carboxyl groups that can be esterified with methyl groups, pectins are classified based on their DE, also known as the degree of methoxylation. Pectins with more than 50% of the carboxyl groups esterified are classified as high methoxyl (HM) and pectins with less than 50% of carboxyl groups esterified are classified as low methoxyl (LM) [82]. DE is one of the most important properties of pectin. For instance, in the gelation mechanism, HM pectin gels at low pH (<3.5) in the presence of 60–65% sucrose, while LM pectin gels in the presence of calcium ions [83].

Beyond technological properties, pectin provides several human health benefits mainly related to the gastrointestinal system. For instance, some of the principal pectin-bioactivities are slowing gastric emptying, improving physical bowel function, reducing glucose and cholesterol absorption, stimulating the immune system, and increasing faecal mass [84]. It has been hypothesized that the covalent cross-linking between phenolic compounds and pectin might confer enhanced antioxidant and antimicrobial activity to this polysaccharide. Table 6 shows some studies on pectin extraction by novel technologies and their bioactive properties. Nancy Picot-Allainet al. [85] reported that pectin extracted from dry lemon peels showed a total phenolic content of 10.20 ± 0.07 mg gallic acid equivalent/g sample and an antioxidant activity of 71.46 ± 1.39 mg TE/g sample with the ABTS method. Regarding the degree of esterification (87.19–92.6%), the lemon peel pectin produced in this study can be categorized as high-methoxyl pectin. Furthermore, the same study also reported inhibition against pancreatic cholesterol esterase, pancreatic lipase, and α-glucosidase by pectin from lemon peels. Presentato et al. [86] discovered that lemon pectin obtained via hydrodynamic cavitation of LP waste displayed significant antibacterial activity against *Staphylococcus aureus*, a Gram-positive pathogen that easily contaminates food. An interesting point in this study is that the antibacterial effect of the pectin extracted from LP waste was superior to that of commercial citrus pectin. However, research studies proving information on this topic are still scarce. Nuzzo et al. [87] showed that lemon pectin ‘’IntegroPectin’ had good results in terms of antioxidant activity (ORAC assay: 122,200 μmol TE/100 g). The antioxidant capacity of pectin can be attributed to its capability to chelate metal ions, and it is influenced by the raw material, the method of extraction, and the degree of esterification of the pectin [81]. Tinh et al. [8] studied the ameliorative effects of LP powder on intestinal inflammation and barrier defects in dextran sulfate sodium (DSS)-induced colitic mice. It was possible to conclude that LP powder (rich in dietary fibers) reduced intestinal damage through the protection of tight junction barriers and suppressed an inflammatory reaction in colitic mice. These results suggest that acetate and n-butyrate produced from the microbial metabolism of dietary fibers in LP powder contributed to reducing colitis. Regarding the bioactivities of pectin, few results have been reported, so this is an interesting topic to be investigated in the future. Taking into account the highest pectin yields reported in the presented studies, a new functional ingredient rich in pectin, which can be obtained in large quantities and at low cost, is a good strategy for valorizing the waste obtained from the citrus juice industry.

## 4. Applications of Lemon Bioactive Fractions in Food Preservation

### 4.1. Direct Incorporation in Food Products

Over the last decade, significant research attention in food fields has focused on addressing the current market demands of consumers by creating new, sustainable, alternative, healthier foods [94]. For this reason, the application of a circular economy in food systems represents a key strategy for the future. Consequently, most studies focus on improving the opportunity to extract bioactive fractions from by-products and incorporate part of them directly in the production of new functional foods (Table 7). Imeneo et al. [95] developed functionalized biscuits enriched with dough containing LP and natural antioxidants extracted from lemon pomace (LPE). These enriched biscuits showed higher phenolic content and antioxidant activity than the control (without lemon fractions) and a longer induction period, which means that the enriched products had a higher intrinsic resistance to lipid oxidation, due to the antioxidant effect exerted by the added LP and the LPE. Moreover, from a sensory point of view, they showed suitable acceptability, in terms of appearance, flavour, and aromatic attributes. Fathy et al. [55] developed a functional ‘’Acidophilus-bifidus-thermophilus (ABT)-Type Synbiotic Yoghurt’’ using citrus peels. The antioxidant activity of ABT yoghurt, fortified with 0.5% LP powder, was 71.10%, which can indicate that the polyphenol profile of LP contributed greatly to the enhancement of the antioxidant activity of the novel ABT synbiotic yoghurt formulated during storage. Ben Hsouna et al. [18] evaluated the incorporation of lemon essential oil in raw beef meat. The application of lemon EO at 0.06 and 0.312 mg/g may open new promising opportunities for preventing the growth of pathogenic bacteria, particularly Listeria monocytogenes. Consequently, these results indicated that the incorporation of lemon processing by-products and their fractions allowed the production of functional foods with improved bioactive properties and improved food safety, and that they can be used as a food additive or flavouring agent.

### 4.2. Edible Films and Coatings

Environmentally friendly technologies aimed at food safety, quality, and easy handling properties have promoted a huge interest in research in coatings for applications, due to their biodegradability, natural protection from the external environment, and potential use as carrier systems for active substances, such as antioxidants, antimicrobials, and flavouring and/or colouring agents [96]. The most important properties of coatings are related to their bioactivity (antioxidant, antimicrobial, and anti-browning) their functionality (barrier to water vapour, oxygen, carbon dioxide, and UV–vis light), their mechanical (tensile stress and elongation at break) and their physical (opacity and colour) properties [97,98]. Preservative edible coatings with antioxidant and antimicrobial properties may delay, reduce, and/or inhibit the growth of microorganisms on the surface of the fruits, thus satisfying consumer demands for safe, nutritious, convenient, and ready-to-use foods [99,100]. In general, base components of coatings are hydrocolloids (proteins or polysaccharides), lipids (waxes, acylglycerols, or fatty acids) or resins [101,102]. Polysaccharides can form a continuous and cohesive matrix, which is related to their chemical structure by the association through hydrogen bonding of their polymeric chains. The main polysaccharides used in edible coatings are chitosan, starch, alginate, modified cellulose, pectin, pullulan, gellan gum, and xanthan gum [103]. The biodegradable films based on polysaccharides are known to be an effective barrier to transference of gases such as O_2_ and CO_2_, although these materials have hydrophilic properties, resulting in a poor water vapour barrier [104,105]. Pectin is an anionic polysaccharide and, as mentioned previously, is strongly represented in lemon peels. Pectin is widely studied for edible coating formulation due to its properties as a food emulsifier, gelling agent, thickener, and stabilizer, and can be found in high concentrations in lemon by-products [69,106]. Furthermore, in recent years, some studies have demonstrated the antioxidant activity of pectin, which is an important bioactivity for the production of pectin-based food additives.

Fresh fruits and vegetables are highly perishable. Approximately 50% fresh produce deteriorates during harvest, handling, transportation, and storage. Edible coatings play a very important role in circumventing this situation and are applied on whole and fresh-cut fruits and vegetables (Table 7). Sessa et al. [107] formulated an edible coating with modified chitosan and nano-emulsified lemon EO to apply to vegetable products. This coating increases antimicrobial activity and prolongs the shelf life of vegetable products. Other authors showed good results with edible coatings. Perdones et al. [20] incorporated lemon EO in a modified chitosan edible coating to apply to strawberries and showed that this coating protected the fruit quality during storage. In this context, edible coatings have been recently proposed in food-related applications due to their excellent barrier to oxygen, aroma preservation, barrier to oil, and good mechanical properties, and because they provide maximum protective effects without much impact on the sensory and organoleptic properties of the food. Edible coatings are currently used in fresh and minimally processed fruits and vegetables [28,108]. 

### 4.3. Antioxidant Dietary Fibres

A low intake of dietary fibres (DFs) contributes to health disorders such as cardiovascular diseases, obesity, and colon cancer [109]. According to the American Dietetic Association, the insoluble/soluble fibre ratio should be 3:1 [110]. The consumption of foods containing a higher proportion of DFs promotes healthier lifestyles, and their regular intake is known to reduce several disorders. Therefore, investigations were carried out to incorporate DF into different formulations offering positive health benefits and functional and technological properties, such as increased water and fat binding and gelling capacity. Adding DF to meat products can also prevent lipid oxidation due to the presence of antioxidant polyphenols. This improves meat products’ emulsion stability, viscosity, rheological properties, and sensory attributes [111]. In this context, some studies have been carried out to incorporate different antioxidant dietary fibre-rich ingredients in food matrices (Table 7). Fernández Ginés et al. [112] investigated the effect of incorporating two types of lemon albedo on cooked bologna sausages: raw albedo (obtained directly from lemons; 65% moisture and 30% fibre content) and cooked albedo (72% moisture and 22% fibre content), at different concentrations (2.5, 5, 7.5, and 10%). The presence of raw albedo (for all the concentrations investigated) increased moisture content. However, when cooked albedo was used, only bolognas with low added albedo concentrations (2.5 and 5%) showed higher moisture content than control samples. Protein and fibre content increased depending on albedo concentration, but no differences were found between bolognas with raw or cooked albedo. The most important result observed in the chemical composition was the fat content decrease and residual nitrite level, which was higher in bolognas with raw albedo than cooked albedo. Soncu et al. [113] designed a study to analyse the effect of lemon fibre addition (2%, 4%, and 6%) on the cholesterol content of low-fat beef burgers. These authors found that lemon fibre addition decrease saturated fatty acid content and reduce the cholesterol content in a concentration-dependent manner. Adding citrus fibre led to a faster relaxation time in low-fat meat, and a significant increase in the proportion of immobilized water. Thus, several additives, including citrus fibre, have been proposed as fat substitutes for their ability to bind water in low-fat meat products. Dietary fibres can be used as functional ingredients/additives to improve meat products’ quality and nutritive properties without changing the flavour. Lemon fibre, as a by-product of the citrus industry, constitutes 25% of the entire fruit mass, and, for this reason, is another source of income for the citrus industry, since it is a cheap, readily available, and natural supplement for the meat industry. Adding ingredients that are a source of insoluble dietary fibres with antioxidant properties could be an opportunity to improve the quality and storage stability of meat products, promoting healthier diets as well, and should be further investigated. 

**Table 7:** Applications of lemon by-products and their bioactive fractions in food preservation.

**By-Products/** **Bioactive Fractions**	**Food**	**Property**	**Packaging**	Results	References
Direct incorporation in food products					
Lemon EO	Minced beef meat	Antimicrobial and antioxidant activity		Lemon EO with preservative effect against Listeria monocytogenes inoculated in minced beef meat.	[18]
Lemon peel powder	ABT synbiotic yoghurt and milk	Antioxidant, antibacterial and probiotic activity		LP improved the antioxidant property of the ABT synbiotic yoghurt and milk; 0.5% LP addition showed higher inhibition against S. aureus, B. subtilis, and E. coli compared to the control; Enhanced the viabilities of probiotic starter cultures with the incorporation of LPs in yoghurt during cold storage.	[55]
Lemon peel and lemon pomace extract	Biscuits	Antioxidant activity		The enriched biscuits with LP and pomace showed higher phenolic content, higher antioxidant activity, longer induction period, and higher intrinsic resistance to lipid oxidation than the control.	[95]
Edible films and coatings					
Lemon EO	Strawberries	Antifungal activity	Chitosan	Delayed ripening with a lower respiration rate was observed in strawberries coated with lemon EO-based chitosan coatings.	[20]
Lemon EO	Vegetable products	Antimicrobial activity	Novel edible coating with modified chitosan and nano emulsified lemon EO	Increases antimicrobial activity and prolongs the shelf life of vegetable products.	[107]
Lemon EO	Strawberries	Antimicrobial activity	Incorporated lemon EO on the modified chitosan edible coating	Protects the storage-keeping quality of strawberries.	[114]
Lemon EO	Biscuit	Antimicrobial activity	Low density polyethylene (LDPE) films	Acts as flavouring films for packaging biscuit, prevents changes in water-vapor permeability and mechanical properties.	[115]
Lemon EO (+ thyme and cinnamon)		Antimicrobial activity	Chitosan	Chitosan film combined with lemon, thyme and cinnamon essential oils provide a new formulation for antimicrobial films.	[116]
Lemon extract	Carrots	Antimicrobial and antioxidant activity	Pectin coating with lemon by-product extract	Improvement microbiological stability of fresh-cut carrots, showing the lowest value of total bacterial (2.58 log CFU g^−1^) Antioxidant activity level (289.49 µM Trolox/100 g).	[117]
Antioxidant dietary fibres					
Lemon fibre/pectin	Cookie	Fat replacer		Incorporating lemon pectin (2.5%, 7% and 10%) reduce 10% of fat material without significant texture differences; the addition of pectin increases water content.	[66]
Antioxidant fibre from lemon albedo	Cooked bologna sausages	Fat replacer		Incorporating two types of lemon albedo: raw and cooked, at different concentrations (2.5%, 5%, 7.5% and 10%) in cooked bologna sausages, decreases the fat content and residual nitrite content.	[112]
Lemon fibre	Low-fat beef hamburgers	Fat replacer		Incorporating lemon fibre (2%, 4% and 6%) to produce low fat beef hamburgers.	[113]
Lemon fibre	Frankfurters	Fat replacer		Lemon fibre led to a faster relaxation time in low-fat frankfurters, and a significant increase in the proportion of immobilized water.	[118]
Lemon fibre/Pectin	Gluten-Free Biscuit	Nutrition fortifier		Replacing 2.5 wt% of the rice flour with lemon pectin obtained from lemon processing waste.	[119]
Lemon fibre from pomace	Dough andMantou (steamed bread)	Nutrition fortifier		The substitution of 3 or 6 g lemon fibre per 100 g flour can produce healthy and acceptable mantou with higher free total phenolic content and antioxidant capacity.	[120]

## 5. Conclusions

Lemon by-products are a rich source of bioactive compounds, especially flavanones, including eriocitrin and hesperidin. Furthermore, lemon by-products are also rich in dietary fibres, notably pectin (soluble fibre) and cellulose (insoluble fibre). In addition, lemon EO, rich in terpenes (D-limonene), is an economically important product due to its flavouring, antioxidant, antimicrobial, and other health-beneficial properties. It is known that conventional extraction methods allow the recovery of these bioactive compounds from lemon by-products. However, high expenditure of time and energy, due to the use of organic solvents, makes conventional methods an unfavourable option. For this reason, non-conventional methods are being increasingly used to recover these bioactive fractions. The type of waste and the molecules to be extracted need to be considered in the selection of the most appropriate technology. Therefore, in response to consumer demand for healthier foods, the use of novel functional ingredients recovered from lemon by-products could be a valid alternative for the formulation of high-value-added products, such as edible preservative coatings with antioxidant and antimicrobial properties and antioxidant dietary fibre powders as fat replacers. In the future, it would be very interesting to valorise these lemon by-products and recover the lemon fractions in an integrated and sustainable process. Three bioactive fractions could be extracted from the same residue: essential oils, phenolic compounds, and dietary fibres. This integrated process would further the implementation of the ‘’zero waste’’ concept, reducing the environmental footprint and achieving a circular economy, to produce sustainable solutions to be applied in the food industry.

## Figures and Tables

**Table 1:** Major terpenoids present in essential oils from lemon by-products.

**By-Product**	Cultivars/Variety	Content of Terpenoids: Identification and Quantification	References
Lemon Peel	*C. limon,* Variety: Fino and Verna (Spain)	Limonene (59.1%), *γ*-terpinene (9.7%), *β*-pinene (5.2%), *β*-bisabolene (3.6%).	[16]
Lemon: Flavedo, Albedo, Juice vesicles and Segment wall	*C. limon,* Eureka variety (China)	Flavedo: Limonene (14.0 mg/g), citral (9.3 mg/g), *γ*-terpinene (4.1 mg/g), *β*-pinene (3.3 mg/g).	[24]
		Albedo: Limonene (2.7 mg/g), citral (0.9 mg/g), *γ*-terpinene (0.4 m/g), *β*-pinene (0.2 mg/g).	
		Juice vesicles: Limonene (1.9 mg/g), citral (0.7 mg/g), γ-terpinene (0.4 m/g).	
		Segment Wall: Limonene (1.1 mg/g), citral (0.4 mg/g), *γ*-terpinene (0.3 m/g), *β*-pinene (0.1 mg/g).	
Lemon Peel	*C. limon,* Four different cultivars: Ovale di Sorrento Sfusato Amalfitano Femminello Cerza Femminello Adamo at different ripening stages (Italy)	Limonene (63.7–76.9%), *β*-pinene (7.7–14.7%), *γ*-terpinene (5.5–10.4%), p-cymene (0.7–1.9%).	[19]
Lemon Peel	*C. limon* (Italy)	Limonene (57.7%), *γ*-terpinene (10.5%), *β*-pinene (9.3%), citronellol (8.2%).	[27]
Lemon Peel	*C. limon,* Five Varieties: Verna, Betera, Eureka, Fino 49, and Fino 95 (Spain)	Limonene (19,760–22,716 mg/kg), *β*-pinene (3757–5011 mg/kg), *γ*-terpinene (3226–3849 mg/kg), *α*-pinene (648.5–797.3 mg/kg), sabinene (611.8–853.0 mg/kg).	[28]
Lemon Peel	*C. limon*, Nanjing Wensenbauer International Trade Co., Ltd. (China)	Limonene (47.3%), *β*-pinene (13.7%), *γ*-pinene (10.6%), trans-citral (4.5%).	[29]
Lemon Flesh	*C. limon*, Juice company (Spain)	Limonene (52.7%), p-cymene (13.7%), *γ*-terpinene (7.4%), *α*-terpinolene (5.1%), *α*-terpineol (4.7%).	[30]
Lemon Peel	*C. limon,* Five Varieties: Lime, Eureka, Volkamer, Limonia, and Red Limonia (China)	Total volatile concentration (12.0–31.6 mg.g^−1^), limonene (5.44–20.5 mg/g; 59.3%–73.3%).	[31]
Lemon Peel	*C. limonum* (Algeria)	Limonene (51.4%), *β*-pinene (17.0%), *γ*-terpinene (13.5%).	[32]
Lemon: (Peel and Pulp)	*C. limon,* Osbeck cv. Femminello Santa Teresa (Italy)	Limonene (60.6%), *γ*-terpinene (13.3%), myrcene (1.9%), *β*-pinene (14.8%), sabinene (3.3%), *α*-pinene (3.1%).	[33]
Lemon Waste	Lemon (*C. limon*) non-conforming fruits (inadequate appearance or size) (Spain)	Limonene (65.1%), *γ*-terpinene (9.7%), *β*-myrcene (1.4%), *β*-pinene (14.5%), sabinene (2.4%), *α*-pinene (1.9%).	[34]
Lemon Peel	*C. limon,* Eighteen cultivars: Sfusato Amalfitano, Ovale di Sorrento, Femminello Fior d’Arancio m 79, Femminello Siracusano m 296, Femminello Dosaco, Femminello S, Femminello Siracusano 2KR, Femminello Scandurra, Iniasel 95, Femminello Continella m 84, CNR L58, Femminello Adamo, Femminello Cerza, Akragas, Selinunte, Segesta, Erice, Kamarina (Italy)	Limonene (72.5–76.4%), *β*-pinene (11.6–18.7%), terpinene (2.9–8.3%), *α*-pinene (1.4–1.5%) and myrcene (0.95–1.1%).	[35]

%: % of Total compounds.

**Table 2:** Extraction technologies and bioactive properties of essential oils from lemon by-products.

By-Product	Lemon Fractions	Extraction Technologies	Extraction Conditions	Antioxidant Properties	Antimicrobial Properties	References
Lemon Flavedo	Essential oils	Microwave-assisted hydrodistillation (MAHD)	525–1800 W, 5–10 min		Growth inhibition at a concentration of 50 ppm for *S. aureus*; Bacteriostatic effect was reached by using 150 ppm (*S. aureus*) and 500 ppm (*Escherichia coli*).	[34]
Lemon Peels	Essential oils	Solvent extraction (Ethanol)	180 min, 4 °C	ABTS assay showed antioxidant effectiveness of peel ethanolic extract of 18 lemon cultivars.		[35]
Lemon: Peels (HD and MAD); Whole fruit (CP)	Essential oils	Hydro distillation (HD); Cold pressing (CP); Microwave accelerated distillation (MAD)	HD: 180 min		The antimicrobial activity for the six microorganisms tested by HD, CP or MAD were similar to MIC.	[40]
			CP: 60 min			
			MAD: 1000 W, 30 min, 100 °C			
Lemon Peels	Essential oils	Steam distillation (SD)	180 min	EO showed 55.1% inhibition of DPPH free radical.	The MIC and MBC value of EO against *S. aureus* was 1.3% and 5%, respectively.	[41]
Lemon Peels	Essential oils	HD; MAHD; Solvent-free microwave extraction (SFME)	HD: 335 W, 120 min	DPPH: IC_50_ values of the EOs extracted by HD, MAHD, and SFME were 44.1, 42.0, and 97.2 mg/mL.		[43]
			MAHD: 1200 W, 15 min			
			SFME: 1200 W, 15 min			
Lemon ‘dried zest’	Essential oils	Aqueous distillation	300 min	EO showed 86.1% inhbition of DPPH free radical.	Antimicrobial activity against saprophytic (*Bacillus subtilis*, *Penicillium chrysogenum*, *Fusarium moniliforme*, *Aspergillus niger*, *Aspergillus flavus*, *Saccharomyces cerevisiae*) and pathogenic microorganisms (*E.coli*, *Salmonella abony*, *S. aureus*, *P.aeruginosa*, *Candida albicans*).	[45]
Lemon Leaves	Essential oils	HD	-		Inhibition against *S.aureus* (32 mm) and *P.aeruginosa* (49 mm).	[42]
Lemon Peels	Essential oils	SD	360 min	DPPH: IC_50_ of 28.91–37.69 mg/mL.	Antimicrobial activity against all tested Gram-positive bacteria and yeasts, and for one Gram-negative (*Pseudomonas fluorescens*).	[46]
Lemon Peels	Essential oils	CP	-	DPPH (0.7 mg/mL); CUPRAC (0.6 mg/mL); Iron chelation (0.7 mg/mL).		[47]

Abb. MIC (Minimum Inhibitory Concentration); MBC (Minimum Bactericidal Concentration); ABTS: 2,2′-azino-bis-3-ethylbenzothiazoline-6-sulfonic acid scavenging activity; DPPH (2, 2-diphenyl-1-picrylhydrazylradical scavenging activity); CUPRAC (CUPric Reducing Antioxidant Capacity).

**Table 3:** Major phenolic compounds (PCs) present in lemon by-products.

**By-Product**	**Cultivars/Variety**	Content of Phenolic Compounds: Identification and Quantification	References
Lemon: Peel, Pulp, Juice, Seeds, and Whole fruit	Five lemon (*C. limon*) cultivars, (China)	Peel: Total PCs: 3.17–4.71 µg/g FW. Phenolic acids: Caffeic: 293.7–741.6 µg/g FW; Chlorogenic: 138.7–527.5 µg/g FW; Gallic: 1.63–90.69 µg/g FW. Flavanones: Eriocitrin: 7.73–49.61 µg/g FW; Hesperidin: 1465–3315 µg/g FW; Hesperetin: 5.79–88.12 µg/g FW.	[9]
		Pulp: Total PCs: 2.43–3.46 µg/g FW. Phenolic acids: Caffeic: 44.67–233.5 µg/g FW; Chlorogenic: 9.28–83.92 µg/g FW; Gallic: 0.95–28.42 µg/g FW. Flavanones: Eriocitrin: ND; Hesperidin: 525.3–1419 µg/g FW; Hesperetin: ND.	
		Juice: Total PCs: 0.29–0.52 µg/g FW. Phenolic acids: Caffeic: 17.83–128.4 µg/g FW; Chlorogenic: 2.70–22.1 µg/g FW; Gallic: 0.38–7.62 µg/g FW; Flavanones: Eriocitrin: ND; Hesperidin: 105.5–210.3 µg/g FW; Hesperetin: 0.83–4.7 µg/g FW.	
		Seeds: Total PCs: 2.1–3.4 µg/g FW. Phenolic acids: Caffeic: 9.31–116.8 µg/g FW; Chlorogenic: 10.11–124.4 µg/g FW; Gallic: 3.50–11.95 µg/g FW. Flavanones: Eriocitrin: 6.7–150.9 µg/g FW; Hesperidin: 10.3–49.9 µg/g FW; Hesperetin: ND.	
		Whole fruit: Total PCs: 1.63–3.04 µg/g FW. Phenolic acids: Caffeic: 127.3–389.2 µg/g FW; Chlorogenic: 1.6–3.0 µg/g FW; Gallic: 1.6–9.0 µg/g FW. Flavanones: Eriocitrin: 0.6–19.6 µg/g FW; Hesperidin: 889.8–9260 µg/g FW; Hesperetin: 1.5–24.5 µg/g FW.	
Lemon Peel and Pulp	*C. limon* (Osbeck cv. Femminello Santa Teresa) (Italy)	Peel: Flavonoids: Vicenin-2: 1.38 mg/100g FW, Diosmetin 8-C-glucoside or 6-C-glucoside: 2.08–2.17 mg/100g FW, Eriocitrin/Neoeriocitrin: 35.4 mg/100g FW, Narirutin/Naringin: 0.73 mg/100g FW, Hesperidin/Neohesperidin: 24.9 mg/100g FW, Total Flavonoids: 66.7 mg/100g FW.	[33]
		Pulp: Flavonoids: Vicenin-2: 3.68 mg/100g FW, Lucenin-2 4′-methyl ether: 4.15 mg/100g FW, Diosmetin 8-C-glucoside or 6-C-glucoside: 2.6–4.9 mg/100g FW, Eriocitrin/Neoeriocitrin: 53.5 mg/100g FW, Narirutin/Naringin: 7.8 mg/100g FW, Hesperidin/Neohesperidin 91.1 mg/100g FW, Total Flavonoids: 168 mg/100g FW.	
Lemon Peel	*C. limon*, juice production company (Tunisia)	Phenolic acids: Hydroxybenzoic acid hexose: 987.9 μg/g DM, Dimer of caffeic acid-O-hexoside: 263 μg/g DM, Sinapic acid hexoside I: 208.6 μg/g DM. Flavonols: Limocitrol-O-glucoside-HMG: 18.3 μg/g DM, Rutin: 14.4 μg/g DM, Quercetin-rutinoside: 12.3 μg/g DM. Flavones: Diosmin or neodiosmin: 102.6–296.4 μg/g DM, Apigenin-7-O-(malonyl-apyosil)-hexoside: 82.5 μg/g DM. Flavanones: Hesperidin: 1234.8 μg/g DM, Neohesperidin: 950.8 μg/g DM, Eriocitrin: 955.4 μg/g DM, Diosmetin 6,8-di-C-β-glucoside: 51.7 μg/g DM.	[50]
Lemon: Peel, Pulp, and Whole fruit	*C. limon*, Interdonato cultivar (Turkey)	Peel: Total PCs: 251.1 mg/100g. Phenolic acids: Ferulic: 32.6 mg/100g; Caffeic: 23.0 mg/100g; Chlorogenic: 20.9 mg/100g.	[51]
		Pulp: Total PCs: 78.6 mg/100g. Phenolic acids: Ferulic: 19.6 mg/100g; Caffeic: 9.9 mg/100g; Chlorogenic: 8.1 mg/100g.	
		Whole fruit: Total PCs: 174.4 mg/100g. Phenolic acids: Ferulic: 28.1 mg/100g; Caffeic: 16.8 mg/100g; Chlorogenic: 15.5 mg/100g.	
Lemon Peel	*C. limon*, local store (Brasil)	Phenolic acids: p-coumaric acid, Dihydroferulic acid. Flavanones: Diosmetin-6.8-di-C-glucosid, Apigenin 6,8-di-C-glucoside, Eriocitrin, Chrysoeriol 6,8-di-C-glucoside, Vitexin 2′′-xyloside, Neodiosmin, Rhoifolin 4-glucoside, Neoeriocitrin, Quercetin-3-O-neohesperidoside, Luteolin-neohesperidosidose, Diosmetin-7-O-rutinoside diosmin, Kaempferol-3-O-Rutinose, Diosmetin 8-C-glucoside, Rhoifolin, Isorhamnetin-3-O-neohesperidoside, Limocitrin-neohesperidoside, Hesperidin.	[52]
Lemon Peel	*C. limon*, local suppliers (China)	Flavanones: Eriocitrin: 27.7 mg/g DM; Hesperidin: 24.5 mg/g DM; Polymethoxyflavones: Sinensetin: 0.7 mg/g DM; Nobiletin: 0.3 mg/g DM.	[53]
Lemon Peel	Edible lemons (*C. limon*), local market (Spain)	Flavanones: Eriocitrin, Eriodictyol, Neoeriocitrin, Naringin, Hesperidin, Hesperetin, Neohesperidin; Flavones: Apigenin, Luteolin, Homoorientin, Orientin, Vitexin, Diosmetin, Rhoifolin, Diosmin, Neodismin; Flavanols: Quercetin, Rutin, Limocitrin, Spinacetin.	[54]
Lemon Peel	*C. limon*, local market (Egypt)	Benzoic acid: 758.7 μg/g DM, p-Hydroxybenzoic acid: 281.4 μg/g DM, Chlorogenic acid: 127.3 μg/g DM, Myricetin: 153.4 μg/g DM, Quercetin: 364.2 μg/g DM, Rutin: 181.1 μg/g DM, Naringin: 269.9 μg/g DM, Total: 2534.1 μg/g DM.	[55]

Abb. FW: Fresh weight; DM: Dry matter; ND: Not detected. Total phenolic was expressed as gallic acid equivalents (GAE).

**Table 4:** Extraction technologies and bioactive properties of phenolic compounds from lemon by-products.

By-Product	Lemon Fractions	Extraction Technologies	Extraction Conditions	Antioxidant Properties	Antimicrobial Properties	References
Lemon: Peel, Pulp, Juice, Seed, and Whole fruit	Phenolic compounds	Methanolic extraction	720 min	Peel: DPPH: 1.08–8.20%. ABTS: 8.65–14.40 mM. FRAP: 1.62–6.60 mM.		[9]
				Pulp: DPPH: 4.00–7.29%. ABTS: 0.94–3.85 mM. FRAP: 0.37–1.85 mM.		
				Juice: DPPH: 0.22–0.59%. ABTS: 0.42–0.71 mM. FRAP: 0.07–0.71 mM.		
				Seed: DPPH: 0.50–4.01%. ABTS: 7.74–11.97 mM. FRAP: 2.30–3.40 mM.		
				Whole fruit: DPPH: 3.10–7.96%. ABTS: 8.79 -13.09 mM. FRAP: 1.15–3.65 mM.		
Lemon Peel	Phenolic compounds	UAE	325 W, 17 min	DPPH: 43.16 mg TE/g DM; ABTS: 18.95 mg TE/g DM; FRAP: 74.95 mg TE/g DM; CUPRAC: 29.35 mg TE/g DM.		[53]
Lemon Peel	Phenolic compounds	Maceration (M) and ultrasound-assisted extraction (UAE)	M: 30 min, 37 °C	DPPH activity using maceration was 22.46% and 39.73% with UAE.		[58]
			UAE: 30 min, 37 °C			
Lemon Albedo and Flavedo	Phenolic compounds	Pulsed Electric Fields (PEF)	0–300 μs, 3–9 kV/cm	Samples with higher phenol content rate showed higher antioxidant activity.		[59]
Lemon Peel	Phenolic compounds	Hydro-ethanolic extraction	120 min, 60 °C		Inhibition against *Aspergillus flavus* of 13.51% after 7 days of incubation.	[63]
Lemon Peel	Phenolic compounds	UAE, Microwave-assisted extraction (MAE) and conventional solvent extraction (CSE)	UAE: 5–20 min, room temperature	DPPH: IC_50_: UAE (268.24 µg GAE/mL); MAE (203.59 µg GAE/mL); CSE (298.82 mg GAE/mL).Higher antioxidant activity for the MAE extracts.		[60]
			MAE: 300–600 W, 1.5–4 min			
			CSE: 120 min, 60 °C			
Lemon Peel	Phenolic compounds	Soxhlet and UAE	Soxhlet: 960 min, 65 °C	The antioxidant activity of samples extracted by UAE was 1.5 to 2.0 times more in comparison to Soxhlet extraction.		[64]
			UAE: 200W, 30–50 min, 40–60 °C			
Lemon Peel	Phenolic compounds	High-pressure extraction	300–500 MPa, 3–10 min	DPPH: 80.93 mg TE/100 g.	LP extract at 0.6 and 0.3 mg/mL concentrations demonstrated antibacterial activity against 16 microorganisms.	[65]
Lemon extract (peel, bagasse, and seed)	Phenolic compounds	Metanolic extraction	-	Antioxidant activity of 74%.		[66]

Abb. DPPH (2, 2-diphenyl-1-picrylhydrazylradical scavenging activity); ABTS (2,2′-azino-bis-3-ethylbenzothiazoline-6-sulfonic acid scavenging activity); FRAP (Fluorescence Recovery After photobleaching); CUPRAC (CUPric Reducing Antioxidant Capacity); ORAC (Oxygen Radical Absorbance Capacity); GAE (Gallic Acid Equivalents); TE (Trolox Equivalents).

**Table 5:** Dietary fibre (DF) composition of lemon by-products.

By-Product	Cultivars/Variety	Content of Dietary Fibre	References
Lemon Peel	*C. limon* from Pokka Sapporo Food and Beverage (Japan)	Total DF: 47.1%; Insoluble DF: 34.8%; Soluble DF: 12.3%.	[8]
Lemon Powder with (Pulp, seed, and peels)	*C. limon*, juice production company (Tunisia)	Total DF in final powder (pulp, seed an peels): 78.68 g/100 g DM.	[50]
Lemon: Peel, pulp, and whole fruit	*C. limon*, Interdonato cultivar (Turkey)	Peel: Total DF: 64.64 g/100g.	[51]
		Pulp: Total DF: 28.29 g/100g.	
		Whole fruit: Total DF: 37.88 g/100g.	
Lemon: Peels and Peeled	*C. limon,* local farmer (Spain)	Peels: Total DF: 2.49 g/100g of FW; Insoluble DF: 67%; Soluble 33%.	[68]
		Peeled: Total DF: 1.31 g/100g of FW; Insoluble DF: 67%; Soluble: 33%.	
Lemon: Peels and Pulp	*C. limon*, from commercial orchar (Spain)	Peels: Pectin: 13% of DW; Lignin: 7.56% of DW; Cellulose: 23.06% of DW; Hemicellulose: 8.09% of DW.	[73]
		Pulp: Pectin: 22.53% of DW; Lignin: 7.55% of DW; Cellulose: 36.22% of DW; Hemicellulose: 11.05% of DW.	
Lemon Peels	*C. limon* (Eureka and Fino 49), juice extraction (Chile)	Eureka: Total DF: 60.1 g/100g DW; Insoluble DF: 50.9 g/100g DW; Soluble DF: 9.2 g/100g DW. Fino 49: Total DF: 68 g/100g DW; Insoluble DF: 62 g/100g DW; Soluble DF: 6.3 g/100g DW.	[74]
Lemon Pomace	*C. limon,* juice industry (Spain)	Total DF: 72.4 8 g/100g DW; Insoluble DF: 14.58 g/100g DW; Soluble DF: 57.8 g/100g DW.	[75]
Lemon: Defatted seed	*C. limon* (Kütdiken), food industry (Turkey)	Total DF: 86.1 g/100g DW; Insoluble DF: 79.6 g/100g DW; Soluble DF: 6.5 g/100g DW.	[76]

Abb. Insoluble and Soluble DF (%): % of Total DF; DW: Dry weight; FW: Fresh weight.

**Table 6:** Extraction technologies and bioactive properties of pectin from lemon peels.

By-Product	Lemon Fractions	Extraction Technologies	Extraction Conditions	Yield (%)	Degree of Esterification (%)	Bioactive Properties	References
Lemon peel	Pectin	Microwave assisted extraction (MAE)	300–700 W, 1–3 min	Citric acid: 5.80%–25.00%	1.2–35.1%		[83]
Lemon peel	Pectin	Acid hydrolisis	180 min, 85 °C	Hydrochloric acid: 27%–39%	87.19–92.6%	Antioxidant activity of 71.46 ± 1.39 mg TE/g sample in ABTS method.	[85]
Lemon peel	Pectin	Hydrodynamic cavitation	-			Antibacterial activity against *S.aureus*.	[86]
Lemon peel	Pectin	Hydrodynamic cavitation	-			Antioxidant activity: ORAC values are remarkably high for ‘’IntegroPectin’’ (122,200 μmol TE/100 g).	[87]
Lemon peel	Pectin	Acid hydrolisis	30–60 min, 60–80 °C	Nitric acid: 17.4%–46.4% Citric acid: 21.4%–76.0%	58.62% 38.46%		[88]
Lemon peel	Pectin	Acid hydrolisis	60 min, 40–90 °C	Citric acid: 36.71%	1.50%		[89]
Lemon peel	Pectin	Ultrasound assisted extraction (UAE) and (MAE)	UAE: 360–600 W, 1–3 min	UAE: Nitric acid: 7.19%–10.11% UAE: Hydrochloric acid: 5.97%–8.60% MAE: Nitric acid: 9.71% MAE: Hydrochloric acid: 7.31%	50.51% 51.13% 50.65% 51.08%		[90]
			MAE: 15–45 min, 60–75°C				
Lemon peel	Pectin	Acid hydrolisis	240 min, 80 °C	Citric acid: 30.6%	18.30–58.06%		[91]
Lemon peel	Pectin	Acid hydrolisis	90 min, 80–90 °C	Hydrochloric acid: 1.38%–2.22%	88.6%		[92]
Lemon waste	Pectin	MAE	600 W, 60–80 min, 100 °C		24–40%		[93]

Abb. ORAC (Oxygen Radical Absorbance Capacity); ABTS (2,2′-azino-bis-3-ethylbenzothiazoline-6-sulfonic acid scavenging activity); TE (Trolox Equivalents).

## Data Availability

The data presented in this study are available on request from the corresponding author.
